# Unheard voices: outcomes of tertiary care for treatment-refractory psychosis

**DOI:** 10.1192/pb.bp.112.042598

**Published:** 2014-04

**Authors:** S. Neil Sarkar, Derek K. Tracy, Maria-Jesus Mateos Fernandez, Natasza Nalesnik, Gurbinder Dhillon, Juliana Onwumere, Anne-Marye Prins, Karen Schepman, Tracy Collier, Thomas P. White, Anita Patel, Fiona Gaughran, Sukhwinder S. Shergill

**Affiliations:** 1 Central and North West London NHS Foundation Trust; 2 Cognition, Schizophrenia and Imaging Laboratory, Department of Psychosis Studies, Institute of Psychiatry, King’s College London; 3 Oxleas NHS Foundation Trust, London; 4 South London and Maudsley NHS Foundation Trust; 5 Health Service and Population Research Department, Institute of Psychiatry, King’s College London

## Abstract

**Aims and method** In up to a quarter of patients, schizophrenia is resistant to standard treatments. We undertook a naturalistic study of 153 patients treated in the tertiary referral in-patient unit of the National Psychosis Service based at the Maudsley Hospital in London. A retrospective analysis of symptoms on admission and discharge was undertaken using the OPCRIT tool, along with preliminary economic modelling of potential costs related to changes in accommodation.

**Results** In-patient treatment demonstrated statistically significant improvements in all symptom categories in patients already identified as having schizophrenia refractory to standard secondary care. The preliminary cost analysis showed net savings to referring authorities due to changes from pre- to post-discharge accommodation.

**Clinical implications** Despite the enormous clinical, personal and societal burden of refractory psychotic illnesses, there is insufficient information on the outcomes of specialised tertiary-level care. Our pilot data support its utility in all domains measured.

In about a quarter of patients with schizophrenia who adhere to treatment, the illness proves refractory to standard treatment protocols.^1,2^ Up to 50% of patients do not respond to clozapine, and there is a lack of novel pharmacological treatments.^3,4^ Various national and international treatment-refractory psychosis guidelines exist:^1,5-7^ most promote a pragmatic approach to prescribing and patient care. Clinical practice, for various reasons, often deviates from guideline recommendations, and one recent large study^8^ demonstrated an average of five different antipsychotics and a mean delay of 4 years before clozapine is commenced in routine clinical practice.

The National Institute for Health and Care Excellence (NICE) advocates the use of tertiary services for treatment-refractory illness. The Department of Health has set out guidelines for specialised services in mental health^9^ which acknowledge that ‘centres of expertise [which] concentrate skills and experience’ are necessary, and offer general treatment-refractory and specific refractory psychosis criteria (Box 1). However, there is a lack of outcome data from specialist tertiary service in schizophrenia, despite the prevalence and burden of treatment resistance. This work aimed to explore outcome measures of one such in-patient service: assessing clinical change from admission to discharge, with preliminary modelling of potential cost-effectiveness. Although data on tertiary care in psychosis are limited, we predicted that admission to the National Psychosis Unit would result in multidomain clinical improvements and this would prove cost-effective.

## Method

The clinical notes of patients admitted to the National Psychosis Unit between 2001 and 2007 were collated for retrospective analysis using the OPCRIT system. This is a reliable and well-validated tool utilising an electronic checklist of psychopathological symptoms that are scored, with algorithms for clinical diagnosis.^10^ The notes on admission to, and discharge from, the National Psychosis Unit were assessed to give comparative OPCRIT scores for each time point. Of 182 sequential notes, 153 had sufficiently detailed clinical information at the time of admission and of discharge for such assessment and accurate completion of OPCRIT scores at the two time points to be made. All patients, 86 male and 67 female, met ICD-10 criteria for a primary diagnosis of schizophrenia, with 36 having a co-existent Axis II diagnosis: 36 had a lifetime history of alcohol dependence or harmful use (24 at the point of admission); 49 a lifetime history of cannabis dependence or harmful use (37 at the point of admission); and 31 a lifetime history of harmful use or dependency on other substances (23 on admission). The mean age on admission was 33 years (s.d. = 10.9), and mean length of stay on the unit was 254 days (s.d. = 169). Thirty patients had their first psychotic episode before the age of 16; in 53 patients it occurred between 17 and 20 years old; in 29, between 21 and 25 years; in 20, between 26 and 35; and 12 had their first episode after the age of 35. At the time of admission, 21 patients were on clozapine monotherapy and 55 patients were taking clozapine with a second antipsychotic.

**Box 1** Proposed criteria for complex and/or refractory disorder services generally, and psychosis services specifically

Generic complex/refractory criteria

- Diagnostic uncertainty hampering treatment
- Persistently high symptom burden
- Significant impact on functioning
- Persisting (>2 years) pattern of incapacity despite appropriate treatment
- Multiple comorbidities increasing likelihood of chronicity
- Need for specialised treatments (e.g. transcranial magnetic stimulation)
- In-patient stay >6-12 months

Specific to a psychosis centre

- Failure to respond adequately (or tolerate) two antipsychotics (at least one atypical)
- Attempted adequate trial of clozapine, usually for a minimum of 6-9 months
- Appropriate psychological therapies such as cognitive-behavioural therapy and family interventions should have been attempted

Abridged and amended from Department of Health guidelines.^9^

To evaluate whether there was a significant difference between admission and discharge scores, paired-samples *t*-tests were conducted on the scores for appearance and behaviour, speech and form of thought, affect and associated features, abnormal beliefs and ideas, abnormal perceptions, and total clinical score. Significance was ascribed according to a false-discovery rate (FDR) corrected *P*-threshold of 0.05 having corrected for the number of variables tested.

Sufficient data were available to allow a preliminary analysis of the costs of the pre-admission and post-discharge social care for 96 of the patients admitted. Costs were based on the patient’s clinical setting immediately prior to admission *v*. immediately post-discharge, which were categorised as home, residential placement, residential rehabilitation unit, hospital in-patient unit and psychiatric intensive care unit. Costs associated with each setting were estimated by extrapolating baseline data from a previous local study of people with schizophrenia in equivalent settings.^11^ That study obtained costing estimate data from various (UK) sources and involved: social security benefit rates from the UK Department for Work and Pensions; police contact costs; specialist education services costs based on data from the Chartered Institute of Public Finance and Accountancy; medication costs from data in the Joint Formulary Committee; and health and social care service costs based on national estimates - for references see Patel *et al*.^11^ Costs in this current study were standardised to one-year periods and updated to 2011/2012 prices.

## Results

### Clinical outcomes from admission to discharge

**Fig 1:**
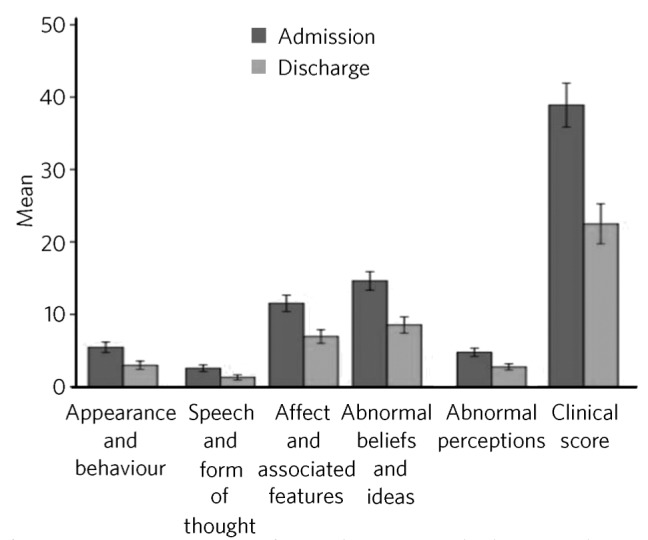
Mean OPCRIT scores from admission to discharge, with 95% confidence interval error bars.

Admission and discharge OPCRIT scores were obtained for all 153 case notes. There was a statistically significant decrease in symptom scores in all domains between admission and discharge (Fig. 1: times 1 and 2 respectively). Significant improvement was seen in: appearance and behaviour (*t*_(152)_ = 7.70, *P*-FDR-corrected = 9.8×10-12); speech and form of thought (*t*_(152)_ = 7.20, *P*-FDR-corrected = 1.9×10-10); affect and associated features (*t*_(152)_ = 11.53, *P*-FDR-corrected = 1.0×10-21); abnormal beliefs and ideas (*t*_(152)_ = 11.67, *P*-FDR-corrected = 4.4×10-22); abnormal perceptions (*t*_(152)_ = 8.63, *P*-FDR-corrected = 4.7×10-14); and global clinical score (*t*_(152)_ = 12.72, *P*-FDR-corrected = 5.6×10-25). A breakdown of OPCRIT scores by pre- and post-admission residency is shown in Box 1. Of note, at the time of discharge, 16 of those admitted on clozapine had had their dose increased, and an additional 63 patients had been commenced on this drug.

### Cost analysis

At discharge, the majority of patients moved to the same (44.8%) or lower- (also 44.8%) intensity setting as compared with their pre-admission setting (Table 1). There was an estimated average saving of £20 929 per person per year between pre-admission and post-discharge accommodation costs. The greatest savings were for those who came from the highest-intensity setting, at an estimated average of £41 358 per person, because many of these moved to a lower-intensity setting post-discharge. Those who moved to a lower-intensity setting (*n* = 43) had a higher National Psychosis Unit admission cost (£143 493 *v*. £98 020; *P*-value from *t*-test 0.009) than those who returned to the same or a higher-intensity setting. They also had a greater OPRIT Mental State Examination score improvement (19 *v*. 9 points; *P* = 0.002).

**Table 1:** Patient admission and discharge residencies (data available for 96 patients), and mean and standard deviation (s.d.) OPCRIT scores

Accommodation type	Number at admission	OPCRIT score at admission (s.d.)	Number at discharge	OPCRIT score at discharge (s.d.)
Home	25	24.6 (14)	28	11.3 (10)
Residential placement	2	18.0 (1)	26	11.4 (11)
Residential rehabilitation unit	13	28.5 (11)	21	17.5 (12)
In-patient/PICU	56	33.9 (15)	21	31.6 (21)
Overall	96	30.4 (15)	96	17.2 (16)

PICU, psychiatric intensive care unit.

## Discussion

Research in refractory psychosis has generally focused on specific individual pharmacological, psychological or sociological interventions, and far less work has explored specialist tertiary units. Undoubtedly such atypical sites have many confounders, including staff make-up and skills, and a cohort of patients whose psychosis is treatment-refractory. Nevertheless, evaluating the work of centres of proposed excellence is clearly worthwhile. Works by Nirodi *et al*,^12^ Ker & Anderson^13^ and Shepherd *et al*^14^ describe the difficulties and rationale for tertiary services more broadly, particularly for treatment-resistant depression, and some of these arguments can be equally considered for refractory psychosis. The nature of commissioning and costing of services in the UK and a push towards primary care management of common disorders mitigate against specialist services. Furthermore, a culture of senior clinicians feeling variously that they ‘should’ know how to manage ‘difficult’ cases, clinical insecurity or clinical overconfidence might be barriers to obtaining a second opinion. However, tertiary care can be argued to afford three broad advantages. First, ‘general’ psychiatrists cannot realistically remain experts in all conditions and with the most recent research developments, nor will they necessarily have the multidisciplinary resources to implement them. Second, specialist services can act as an expert resource for consultation and in training generalists, aiding clinical development and confidence. Third, the academic links typically found in centres of excellence facilitate evaluation and more rapid integration of novel therapeutic developments.

### Study limitations

There are a few caveats that require highlighting when reviewing a naturalistic data-set with no control group. Primary issues are that a full data-set was not available for all those admitted; comparisons were only possible at two time points in individuals; patients are admitted when chronically unwell and failing to respond to treatment, and may potentially be unlikely to be offered medically recommended discharge until some improvement is shown; adherence to medication may be better in a closely monitored in-patient unit. Nevertheless, there was evidence for a statistically significant improvement, across all clinical domains, in a cohort of patients with psychosis deemed unresponsive to standardised secondary care. Although the OPCRIT is considered a reliable and well-validated tool, its design was primarily for extracting diagnostic information from case notes. Its use in retrospective data collection and as a mechanism for scaling symptom severity is open to challenge^15^ and one cannot exclude the inadvertent biases introduced during the rating process. Two of the authors (S.N.S. and G.D.), both psychiatrists, collected the OPCRIT data: they undertook interrater reliability training, and a test-retest on a random sample of ten sets of notes showed good reliability. The costings model is inevitably somewhat crude, but the factors utilised were those that were identified from earlier cost-effectiveness studies and consistently noted in the patient records. Nevertheless, future work might undertake a more rigorous exploration of pre- and post-treatment costs as well as longer-term follow-up of clinical and financial outcomes over several years. The patient/demographic variables, illness variables (such as duration of illness, number and length of episodes), and clinical input variables (duration of admission, number and nature of treatments trialled) were not explored as data variables. Inevitably with the well-established issue of responder bias, the findings of any survey with low total numbers must be interpreted judiciously.

### Challenges to tertiary services

There are several logistical challenges in provision of specialist services, particularly where these are geographically distant from the patient’s home, making the necessary communication with carers and locality more challenging. There are some approaches which can mitigate these to some degree; for example, the National Psychosis Unit holds monthly carers’ groups with an aim of providing access to support and information and to discuss their lived experiences. In this context, the care programme approach provides an essential framework for regular communication between the locality teams, carers and the specialist centre and, in our experience, this forum is viewed very positively by all participants. Finally, the National Psychosis Unit is unusual in being integrated with the Psychosis Clinical Academic Group at the Institute of Psychiatry, King’s College London, enabling very close links with active researchers and access to novel treatments.^16-18^ State-of-the-art investigations^19-21^ and therapeutic monitoring are easily available, as are the necessary liaison with specialised pharmacy, haematology and cardiology. The recovery-based focus is also facilitated by access to a full range of occupational therapeutic and highly specialised psychological therapies for psychosis. Our initial findings support the effectiveness of admission to a specialist in-patient service, but longer-term prospective data are required, particularly looking at the specific patient and clinical input factors that might affect outcomes.^22-24^
