# Unheard voices: outcomes of tertiary care for treatment-refractory psychosis — ADDENDUM

**DOI:** 10.1192/bjb.2019.40

**Published:** 2019-08

**Authors:** S. Neil Sarkar, Derek K. Tracy, Maria-Jesus Mateos Fernandez, Natasza Nalesnik, Gurbinder Dhillon, Juliana Onwumere, Anne-Marye Prins, Karen Schepman, Tracy Collier, Thomas P. White, Anita Patel, Fiona Gaughran, Sukhwinder S. Shergill

There was an oversight regarding the acknowledgement of funding from the European Research Council, which was a contractual obligation for the funding. Professor Sukhwinder S. Shergill was funded by an ERC Consolidator Award.
